# Comparative Analysis of Quality of Life of Patients with Dermatological Problems: Teledermatology Versus Face-to-Face Dermatology

**DOI:** 10.3390/healthcare10112172

**Published:** 2022-10-30

**Authors:** Remedios Lopez-Liria, Antonio Lopez-Villegas, Maria Angeles Valverde-Martinez, Mercedes Perez-Heredia, Francisco Antonio Vega-Ramirez, Salvador Peiro, Cesar Leal-Costa

**Affiliations:** 1 Health Research Centre, Department of Nursing, Physiotherapy and Medicine, University of Almeria, Carretera del Sacramento s/n, La Cañada de San Urbano, 04120 Almeria, Spain; 2 Research Unit, Poniente University Hospital, El Ejido, 04700 Almeria, Spain; 3 Research Management Department, Primary Care District Poniente of Almería, El Ejido, 04700 Almeria, Spain; 4 Research Team Hum-498, Torrecardenas University Hospital, 04009 Almeria, Spain; 5 Health Services Research Unit, Foundation for the Promotion of Health and Biomedical Research of Valencia Region (FISABIO), 46020 Valencia, Spain; 6 Nursing Department, University of Murcia, 30003 Murcia, Spain

**Keywords:** primary care, health-related quality of life, teledermatology, telemedicine, dermatology

## Abstract

The health-related quality of life (HRQoL) of the patients cared for with teledermatology (TD) services was analyzed as compared with face-to-face dermatology (F-F/D) at the hospital. This study was a controlled, non-blinded, intra-level, and multicenter randomized clinical trial, with a 6-month follow-up. A total of 450 patients were randomly assigned to two different groups. The Spanish version of the generic EuroQol-5-dimensions-5-Levels (EQ-5D-5L) questionnaire and the specific Skindex-29 questionnaire were used at 0 and 6 months. The number of primary care visits (2.24 TD; 1.68 F-F/D) and number of hospital visits (0.01 TD; 1.48 F-F/D) were statistically significant. It was observed that from month 0 onwards, the users included in the F-F/D group self-perceived a lower HRQoL than the users included in the TD group (Skindex-29 total: *p* ≤ 0.00; EQ-5D-5L VAS = *p* ≤ 0.00; EQ-5D-5L utilities = *p* ≤ 0.00). At the end of the study, the patients included in the F-F/D group still obtained lower scores in their perception of HRQoL, as compared to those included in the other type of follow-up (Skindex-29 total: *p* ≤ 0.00; EQ-5D-5L VAS = *p* ≤ 0.00; EQ-5D-5L utilities = *p* ≤ 0.00). TD was an effective diagnosis and follow-up tool. At the end of the study period, the HRQoL of the patients in both groups was significantly higher as compared to their baseline levels. Additionally, both the general and specific HRQoL perceived by the TD patients was higher than the F-F/D group from the start of the study.

## 1. Introduction

Dermatological diseases are the fourth most common of all the human diseases. Although their prevalence varies according to geographical area, they affect more than 50% of the population worldwide, and are some of the main reasons behind primary care (PC) consultation [1,2].

There is a great diversity of skin diseases, which vary according to their severity and symptoms; among others, we find infections, benign tumors, inflammatory diseases, or malignant neoplasms, which cause a significant morbidity and create direct and indirect costs. Skin cancer is one of the most prevalent dermatological afflictions in clinical practice, and its global incidence increases with age [3,4,5]. Its early detection is fundamental, and it is necessary to provide precise diagnoses, based on clinical examination and histopathology, to ensure the efficacy of treatment [6,7,8]. However, diagnostic precision tends to fall on PC doctors, nurses, or auxiliary personnel, given the scarce number of dermatologists [9].

With the digital era, the use of technology in the area of health has become increasingly important [10,11]. Since its appearance in 1995, the use of teledermatology (TD) has increased significantly, and even more so during the COVID-19 pandemic [12]. This monitoring alternative is utilized for the remote diagnosis, assessment, and treatment of dermatological afflictions, to improve the patient’s access to care, increase efficiency, reduce the wait times, and the costs associated with the face-to-face dermatology (F-F/D) modality [12,13]. There are three TD modalities: synchronous, asynchronous, and hybrid [14,15,16]. 

On the other hand, the dermatological afflictions also have an influence on the health-related quality of life (HRQoL), with repercussions on social (isolation), work (limitations for performing specific tasks), economic (direct and indirect costs), emotional (altered emotional reactions), family, and sexual aspects [15,17]. For this, the assessment of the HRQoL of patients with dermatological afflictions is needed, as it is useful for clinical practice and therapy adherence [18,19]. The World Health Organization’s definition of HRQoL is a broad concept that is influenced by physical health, psychological state, social relationships, and personal beliefs in the context of the cultural and value systems in which one lives [20].

The implementation of TD services in many countries has been oriented towards the improvement of the HRQoL of patients. However, it is unknown if there are significant differences with respect to the effectiveness of these monitoring modalities (TD versus F-F/D). The administration and posterior analysis of the information provided by these questionnaires during the anamnesis of the patients has shown their perception of HRQoL at different moments in time during their dermatological illness [21,22,23].

Previous studies have confirmed that TD offers a fast response from the specialist’s consultation, reduces unnecessary travel, early diagnoses, and priority in the care of the most severe cases [11]. Despite these advantages, not many studies have addressed the general or specific perception on HRQoL of users of this alternative modality of care.

The objective of the present study is to analyze the quality of life of patients with dermatological problems, by comparing the teledermatology services with face-to-face (F-F/D) modality at the hospital.

## 2. Materials and Methods

This is a controlled, non-blinded, intra-level (PC and Poniente University Hospital), and multicenter study (all the health centers part of the Western Health District), with a 6-month follow-up. The project was approved by the Research Ethics Committee from Centro-Almería (CEIC-AL) with code 27/2020, and the trial was registered at ClinicalTrials.gov ID: NCT04378296.

### 2.1. Settings and Patients

The study took place at the Western Municipality of Almería (Comarca del Poniente, Andalusia, Spain), with a reference population of 265,000 inhabitants. The experimental group was composed of patients with dermatological problems who were cared for asynchronously (TD), while the control group was composed of patients who were monitored in person at the hospital (F-F/D).

The inclusion criteria were: being older than 18 years old, having a dermatological disease (without excluding by type or classification), and accepting to participate in the study. The exclusion criteria were: having non-dermatological diseases that could affect the study, refusing to participate, or participating in another study.

The study was designed to include the spectrum of skin disease expected in the target population. We collected the patient’s diagnosis into 4 categories: “injuries”, “rash”, “injuries and rash”, or “others” diagnosis. Skin injury was the risk for alteration in epidermis and/or dermis (such as pressure ulcer, irritant dermatitis, inflammatory dermatitis, infection, chronic tissue injury, etc.). Rash was defined as the change of the human skin which affects its color, appearance, or texture (presence of macules papules, pustules…). Rashes are generic terms used by people and doctors to denote the changes in the skin such as skin infections, skin allergies, and skin diseases (e.g., autoimmune diseases, atopic dermatitis or eczemas, acne or rosacea, urticaria and erythema). The “others” category included a range of diseases of the skin and subcutaneous tissues such as skin cancer, sexually transmitted diseases, benign growth, or Lichen simplex chronicus.

**Figure 1:**
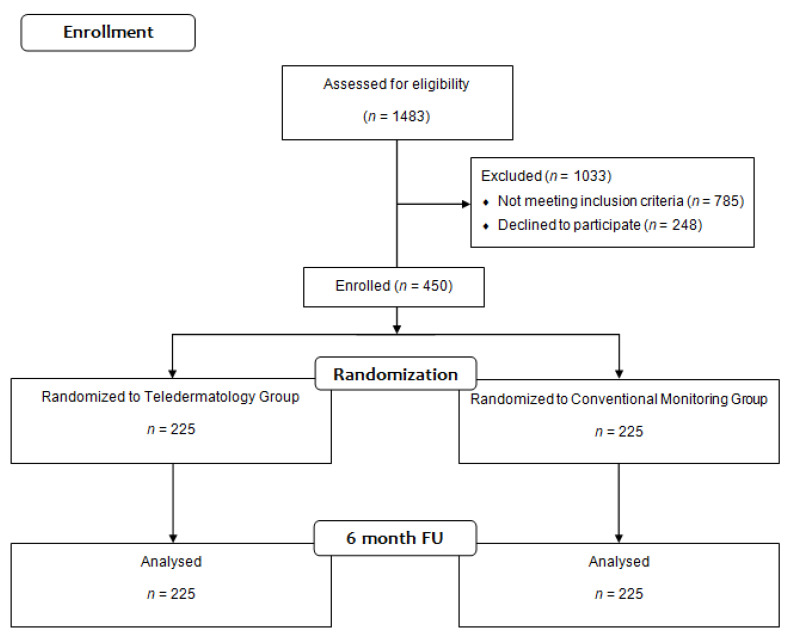
CONSORT 2010 Flow Diagram participants.

Figure 1 below shows the procedure utilized to include the patients and to assign them to either the experimental or the control groups.

The patients were randomly selected in both groups, with systematic random sampling. In this study, a random number was chosen, and inclusion into the group was proposed to one patient out of every three who entered the PC consultation to request dermatological health care. In the case that the patient did not want to be included in the study, inclusion was proposed to the next patient. The number of participants included in each of the follow-up groups was proportionally distributed into each of the Clinical Management Units [24].

### 2.2. Procedure

The TD protocol in this asynchronous modality consists of the patient going to the consultation, where the PC doctor fills out a request form for a teleconsultation in the digital platform “Telederma”, indicating the characteristics and localization of the lesion, previous treatment (if any), medical history, etc. In a period lasting less than 10 days, the patient goes to the appointment at the PC center. The nurse asks for the signed informed consent form previously provided by the doctor (a type of proof established by the Ministry of Health, through which the patient agrees to be attended telematically). Afterwards, the nurse verifies that all the information needed by the doctor is correctly recorded; then, the nurse takes pictures of the skin area using a sequence defined in a protocol for their correct viewing. Independently of the lesions, a panoramic picture is taken less than 1 m away, which clearly shows the area of the body. Then, images with a polarized light are taken with a dermatoscope, and whenever possible, a measurement scale will be included to determine the size of the lesion in case a posterior follow-up is needed. Lastly, the images are saved on the platform. On the upper part of each image, there is a section reserved for “observations”, in which incidences or observations that could be useful for the in-hospital dermatologist, can be described in detail. Lastly, the images are eliminated from the camera before continuing with the following patient.

Normally, within 20 days (in non-severe cases), the patient is given an appointment by the PC doctor to obtain the results, along with the diagnosis, treatment, and other indications the dermatologist may deem important. If a severe pathology is suspected, the timeframe can be reduced between 24 h and 2 days.

For the patients included in the study, a minimum of two assessments were given in person and/or via telephone (at the start of the study, and after 6 months).

The healthcare professionals in this study were: 15 physicians and 7 nurses corresponding to the 5 Basic Health Zones (Adra, Berja, El Ejido, Roquetas de Mar and Vícar) that comprise the Primary Care Service of the Poniente de Almeria Health District. On the other hand, 4 dermatologists and 4 nurses participated in the conventional follow-up performed at the hospital. 

### 2.3. Measurements and Instruments

In this study, the characteristics of the patients (age, sex, diagnosis, origin), as well as the number of visits to the hospital, were analyzed. As the main measurement, the HRQoL of the patient was analyzed at month 0 and after 6 months of follow-up, with the Spanish version of the generic questionnaire, EuroQol-5-dimensions-5-Levels (EQ-5D-5L) [25,26,27]; and as a secondary measurement, the specific quality of life measured with the administration of the Spanish version of the Skindex-29 questionnaire [28,29,30,31].

The Spanish version of the EQ-5D-5L measures mobility, personal care, everyday activities, pain/discomfort, and anxiety/depression [25]. Its responses range between 0 (death) and 1 (the best state of health possible), although negative scores can be used for states “worse” than death. It is the only tool adapted to Spanish, with a high value for its use in cost-effectiveness studies, especially in the assigning of health resources [25]. The Visual Analog Scale (VAS) of the EQ-5D-5L is used to assess between the worst state of health possible (0) and the best state of health possible (100) [26,27]. The advantages of generic instruments lie in their use in making comparisons between diseases and for developing norms for a particular population or medical condition [22,23,26,31].

The dermatological questionnaire of quality of life, Skindex-29 [28,29,30,31,32], is a well-known dermatology-specific instrument that assesses the emotional, functional, and symptomatic dimensions, and offers an overall score. It is expressed in a linear scale, varying from 0 to 100 (with higher scores representing the worst quality of life), with 5 response options [28,29,30].

### 2.4. Statistical Analysis

The basic characteristics of the patients, and the possible differences between groups were analyzed with the Chi-square test (substituted by Fisher’s exact test in case of n < 5 cases) for the qualitative variables, and the differences in means test for quantitative variables. The intra-group differences between the 0- and 6-month assessments were analyzed through the use of the differences in means or differences in ratios for paired data.

The Mann–Whitney U test was used to compare the variables of the EQ-5D-5L questionnaire in both groups, as these were ordinal values. The Mann–Whitney test is a non-parametric test used to compare the median of two independent samples, and to determine the existence of differences between them. It is used as an alternative to the Student’s *t*-test when we cannot assume the normality of these samples.

To compare the EQ-5D-5L VAS at both moments in time (basal and after 6 months), Wilcoxon’s multiple range test was mainly used for dependent samples, as it compares the medians of two samples and determines if there are differences between them.

The Skindex-29 questionnaire includes dichotomous (YES/NO) qualitative items; for the comparison between groups, non-parametric tests were used, and Fisher’s exact test when comparing frequencies in 2 × 2 tables.

The repeated measures mixed effect model were used to discern whether there were any different long-term measurements for each of the groups in the different dimensions of both the EQ-5D-5L and Skindex-29 questionnaires.

The results are presented by including the respective confidence intervals at 95% (95%CI). All the analyses were performed with the statistical package SPSS v.18.0.0 (SPSS Institute, Inc., Chicago, IL, USA).

## 3. Results

The baseline characteristics of each group studied are presented below (Table 1).

The mean age of the patients in both groups was similar, at around 52 years old. The percentage of males and females was also distributed equally. With respect to the EQ-5D-5L questionnaire utilities and VAS, initial statistically significant differences were found between the TD (0.89 and 76.22, respectively) and the F-F/D (0.77 and 68.24) groups.

The origin of the patients was mainly El Ejido (41.11%) and Roquetas del Mar (33.33%). As for ethnicity, significant differences were not found between the groups, with the most numerous groups being: White/Caucasian (TD = 94.4% versus F-F/D = 92%) and Arab (TD = 3.56% versus F-F/D = 3.56%).

The reason for consultation were: injury (62.22% TD; 40.89% F-F/D) or other problems (23.56% in TD and 44% in F-F/D groups) with statistically significant differences. In the TD group, the percentage of patients who had not had any diagnostic test performed was 93.78%, as compared to 67.56% of the F-F/D group; and only 2.67% needed a biopsy in the TD group, compared to 20% of the F-F/D group.

The anatomical location of the skin problem in both groups was similar: head and neck (50.2% TD and 43.3% F-F/D), limbs (18.7% TD and 22.8% F-F/D), trunk (18.5%), and percentages lower than 6% for other body parts.

The treatment was mostly pharmacological (64.9% TD and 44.4% F-F/D), surgical (13.8% TD and 40.9 F-F/D), or a follow-up and evolution was performed (20% TD and 4.4% F-F/D). 

The number of PC visits (2.24 TD and 1.68 F-F/D) and the number of hospital visits (0.01 TD and 1.48 F-F/D) were statistically significant. 

Health-related quality of life (HRQoL): when comparing the results from the EQ-5D-5L at month 0 and after 6 months, statistically significant differences were observed in all the items between both groups, except for the variable PAIN/discomfort after 6 months (Table 2). That is, it was observed that at baseline, differences were found between the groups with respect to HRQoL (the control group started with a worse quality of life, but after 6 months, these differences disappeared with respect to pain/discomfort, as both groups improved their state in this dimension in a similar manner). Although the improvement in the HRQoL was evident in both groups, the control group had a more favorable evolution, as the starting scores were worse than the TD patients.

With respect to the comparison of the EQ-5D-5L questionnaire, and the specific Skindex-29 questionnaire in both assessments (baseline and at 6 months) in each of the groups, Table 3 shows that there were statistically significant differences in all the dimensions. That is, it can be confirmed that there are differences in the HRQoL measured with the Skindex-29 questionnaire in the dimension’s functionality, symptoms, or emotions after 6 months, for both the TD and the F-F/D groups. Differences were also found in the EQ5D VAS and utilities in each of the groups, after 6 months from the first assessment. Therefore, the patients experienced a significant improvement in their HRQoL in both groups after their follow-up either with TD or F-F/D.

Next, the results from each of the items of the Skindex-29 questionnaire are presented, to discover which of these were more relevant with respect to the comparison between the TD group and the F-F/D one in the first assessment, and after 6 months (Table 4).

The items in which statistically significant differences were not found at the baseline between the experimental group and the control group were: “I am worried that my skin disease might be something serious”, “my skin itches”, “I am ashamed of my skin disease”, “I tend to do things alone because of my skin disease”, ”My skin disease causes me to be embarrassed”, “I find my skin disease humiliating”, “My skin disease interferes with my sex life”, and “My skin disease makes me tired”. 

After 6 months, the improvements in quality of life were maintained, without statistically significant differences between the groups in any of the items, except for the following statements:

“I am worried that my skin disease might be something serious”, where a better score was found in the TD group and the percentage of patients who answered “yes” to this statement decreased considerably. For the statement “My skin disease makes me depressed”, a reduction was observed in the TD group, with an increase (3.10%) observed in the conventional group (3.10%). On the other hand, for the statements “I worry that my skin disease will get worse” and “My skin disease makes me angry”, a slight increase was observed in the positive answers with respect to the TD group (0.50 and 2.20%). For the statement “I tend to do things alone because of my skin disease”, a reduction was observed in both groups, but the F-F/D group evolved more favorably, as the percentage of patients who answered “yes” to this question was reduced in a higher percentage. Another aspect that could be highlighted is that in both of the following statements “My skin is irritated” and “My skin is sensitive”, a strong reduction was observed after 6 months in both groups, although this reduction was slightly better in the F-F/D group. As a greater number of patients in this group had answered affirmatively, after 6 months, the difference was greater with respect to the TD group, in which the patients had complained less since the first assessment.

As can be seen in Table 5, there were significant differences in the *p*-values of two of the five evaluated dimensions of the EQ-5D-5L questionnaire (mobility and usual activity) in the three interactions analyzed (groups, time, and time*group).

Likewise, Table 6 shows that there were significant differences in the *p*-values of all the evaluated dimensions of the Skindex-29 questionnaire (symptoms, emotions, functionality, and total) in the three interactions analyzed (groups, time, and time*group). In addition, the F-F/D group had a greater decrease in activity values than teledermatology.

## 4. Discussion

This randomized controlled trial (RCT) reported significant improvement in HRQoL in both TD and F-F/D groups, as evidenced in the mean EQ-5D-5L and Skindex-29 scores after 6 months. Although the groups did not show a similar distribution of clinical baseline characteristics including HRQoL, after the treatment, an increase in HRQoL scores compared to baseline values was observed during the 6-month follow-up, but a different course for separate scales was clear. The descriptive information provided by the Skindex-19 scale is very interesting, as it highlights the specific aspects of the HRQoL that are given more importance by the dermatological patients, or that affect their lives, pointing to aspects such as: “I am worried that my skin disease might be something serious”, “My skin itches”, “I worry that my skin disease will get worse”, “My skin is irritated or sensitive”, or “My skin disease burns or stings”. The quality of life of the F-F/D group after 6 months was greater when compared to the baseline, although the final scores of the quality of life in general terms were worse than the TD patients. On the other hand, it should be pointed out that the patients cared for with TD visited the doctor with a mean of 2.24 sessions as compared to 3.16 sessions of the F-F/D group. This information is considerably similar to a recently published systematic review with eight studies, which provided information on approximately 16,539 patients, indicating that TD proved satisfactory to both patients and professionals, and with lower costs than the face-to-face modality, related with the number of in-person visits to the health professional [15]. Whited’s review [31] points out that the HRQoL is a gap in TD and shows that this intervention results in improved quality of life, and those changes correlated with improvements in disease severity and clinical course.

There are intrinsic and extrinsic factors associated with aging of the skin, which therefore lead to the development of skin lesions. The greater one’s age, the greater the aging of the skin, which justifies the mean age of the patients in our study at 52 years old. On the other hand, although our study showed an equal distribution between men and women, other studies indicate the predominance of the men [33,34,35,36].

The effects that a dermatological disease can have on one’s general appearance can result in the functional and psychological affliction of those who suffer from it [37]. Although the psychological aspect has been largely forgotten by health professionals, at present, mental health has gained the importance it deserves, as it is directly associated with the quality of life of the patient (particularly in psychosocial and emotional domains) and his or her optimal recovery [31,36,37]. Studies such as that by Restrepo et al. [36] show that some patients with dermatological illnesses (psoriasis, vitiligo, hives) suffer from sleep disorders and depression, with negative effects at the personal and work levels.

As indicated in our study, in general terms, there are statistically significant differences with respect to the HRQoL of the patient with a dermatological affliction between the baseline and after six months, with both TD and F-F/D treatment modalities being beneficial. A study conducted in 2013 [38] compared store-and-forward teledermatology with conventional consultation processes to manage the dermatology consultations in 326 patients. Clinically significant improvements were reported for symptoms, emotions, and composite score for both randomization groups, but no significant differences in Skindex-16 scores between treatment groups were found. 

Parsi [39] compared an online care model for follow-up treatment of patients with psoriasis with a conventional in-office model and did not find significant differences in Dermatology Life Quality Index (DLQI) scores between the two groups (*p* = 0.79), a specific questionnaire, that together with the Skindex-29 questionnaire, tends to be the most utilized by the scientific community. Another study in line with these findings is that of Os-Medendorp [40] on patients with atopic dermatitis, which did not find significant differences in HRQoL (measured using the DLQI) or severity and intensity of itching (with the Impact of Chronic Skin Disease on Daily Life’ questionnaire) between individualized e-health compared with usual F-F/D. For Datta [41], HRQoL was determined according to living longer with a dermatological condition versus a shorter life with perfect health. The utility gain among the participants in the TD group (0.03) compared with the gain among those in the conventional group (0.02) was not statistically significant (*p* = 0.50). 

On the other hand, it is important to point out that already authors such as Williams et al. [42] in 2001, when the first TD studies began, showed that patient satisfaction with TD was related to perceived quality of life in a study with 123 adult patients with non-urgent dermatology referrals from PC. As a result, patients reporting a lower HRQoL as measured by the DLQI were more likely to prefer a face-to-face encounter with a dermatologist and to evince anxiety about being photographed. In 2020, Standler et al. [43] analyzed patients’ opinions on TD during the COVID-19 pandemic, including the quality of life (with DLQI). A total of 91 patients took part in their survey. Regarding the DLQI, the minimum was 0 and maximum 27 points, and the participant’s mean was 6.19. They concluded than dermatological care using more modern telemedicine technologies than telephone conferencing is needed to better address patients’ desires. 

In a recently published article, pertaining to this same study, based on patients’ experiences, the results showed [24]: although there were generalized positive experiences of the patients with both follow-up modalities, the TD group indicated having received less information about the diagnosis and they were less involved in the decisions. However, they perceived more confidence in the professional skills of the doctors and described PC institution as better organized.

A number of limitations need to be taken into consideration when evaluating the results of this study: it is necessary for the patients to have similar characteristics in both groups to obtain more evident and reliable results that can be extrapolated to the rest of the population. The generalizability of our findings may be constrained by the demographics of our study population and, the representative skin conditions. In the present study, the largest number possible of patients was used randomly without selecting the groups according to pathologies, which could have influenced the responses of the patients, according to their subjective perception of the severity of their affliction. However, because these data were collected in the context of a randomized clinical trial, any potential bias is more likely to influence absolute values rather than incremental differences. Clinic-based procedure recommendations (e.g., biopsies) were expected to result in an in-person dermatology hospital visit (F-F/D). On the other hand, some patients can feel embarrassed when photographs of their skin lesions are taken, and they were worried about the privacy of the images in a study. This distrust caused that sometimes they decided to decline to participate. Generic instruments do not necessarily capture relevant features or domains that are specific to skin disease, which is the reason why a dermatology-specific questionnaire was also included in a heterogeneous group of skin conditions.

One strength of this study is its conduct in the context of a true site-to-site setting of primary care to dermatology referral. This RCT carries implications for clinical practice that includes interesting results about the HRQoL in TD compared to following a F-F/D regime in a large number of patients, providing detailed and comparatively information, and TD’s impact on patients receiving this care with two international, valid and reliable scales (EQ-5D-5L and Skindex-29).

These results show that both treatment modalities are effective with respect to the HRQoL of patients with dermatological afflictions. Some studies advocate the combination of TD and F-F/D to obtain benefits from both [44]. For this, TD must be part of the toolbox available to the health professional, considering the numerous advantages for the health system from a social and economic perspective.

## 5. Conclusions

At the end of the study period, the HRQoL of the patients included in both groups was significantly higher compared to that observed in the baseline analysis. Additionally, the HRQoL, both generic and specific, perceived by the TD patients, was greater than that of the F-F/D group from the start of the study. TD seems to be a tool that is similarly effective for the diagnosis and follow-up of patients with dermatological pathologies as compared to the F-F/D modality.

## Figures and Tables

**Table 1:** Selected patient baseline characteristics.

	All	Groups		*p*-Value
		Teledermatology (n = 225)	Face-to-Face Dermatology (n = 225)	
Age M(SD)	52.16 (19.97)	52.53 (18.17)	51.78 (21.65)	0.744 ^a^
Women n (%)	240 (53.3%)	131 (58.2%)	109 (48.4%)	0.047 ^b^
EQ-5D utilities (mean) [95 CI]	0.83 [0.81–0.85]	0.89 [0.87–0.91]	0.77 [0.74–0.81]	0.000 ^b^
EQ-5D VAS (mean) [95 CI]	72.23 [70.06–74.40]	76.22 [73.29–79.15]	68.24 [65.10–71.38]	0.000 ^b^
Race/ethnicity n (%)				
White/Caucasian	415 (92.22%)	208 (94.4%)	207 (92%)	0.453 ^b^
Gypsy	6 (1.33%)	4 (1.78%)	2 (0.89%)	
Hispanic/Latino	10 (2.22%)	5 (2.22%)	5 (2.22%)	
Black	3 (0.67%)	0 (0%)	3 (1.33%)	
Arab	16 (3.56%)	8 (3.56%)	8 (3.56%)	
Reason for consultation n (%)				
Injury	232 (51.56%)	140 (62.22%)	92 (40.89%)	0.000 ^b^
Rash	52 (11.56%)	24 (10.67%)	28 (12.44%)	
Injury and rash	14 (3.11%)	8 (%)	6 (2.67%)	
Other	152 (33.78%)	53 (23.56%)	99 (44%)	
Diagnostic tests performed n (%)				
None	363 (80.67%)	211 (93.78%)	152 (67.56%)	0.000 ^b^
Blood test	31 (6.89%)	7 (3.11%)	24 (10.67%)	
Biopsy	52 (11.56%)	6 (2.67%)	46 (20.44%)	
Micro punctures	3 (0.67%)	1 (0.44%)	2 (0.89%)	
Anatomical location of the skin problem n (%)				
Trunk	83 (18.5%)	41 (18.2%)	42 (18.8%)	0.336 ^b^
Limbs	93 (20.7%)	42 (18.7%)	51 (22.8%)	
Head and neck	210 (46.8%)	113 (50.2%)	97 (43.3%)	
Limbs. head and neck	13 (2.9%)	6 (2.7%)	7 (3.1%)	
Trunk and limbs	24 (5.3%)	14 (6.2%)	10 (4.5%)	
Whole body	10 (2.2%)	5 (2.2%)	5 (2.2%)	
Trunk. head and neck	16 (3.6%)	4 (1.8%)	12 (5.4%)	
Treatment n (%)				
Pharmacological	246 (54.7%)	146 (64.9%)	100 (44.4%)	0.000 ^b^
Surgical	123 (27.3%)	31 (13.8%)	92 (40.9%)	
Follow-up and evolution	55 (12.2%)	45 (20%)	10 (4.4%)	
Pharmacological. surgical and follow-up	26 (5.8%)	3 (1.3%)	23 (10.2%)	
Number of Primary Care visits M(SD)	1.96 (0.76)	2.24 (0.65)	1.68 (0.76)	0.000 ^a^
Number of hospital visits M(SD)	0.75 (0.95)	0.01 (0.09)	1.48 (0.85)	0.000 ^a^

^a^ *t*-Student test; ^b^ Chi Squared test.

**Table 2:** Changes in HRQoL between baseline and 6 months after the first visit, across EQ-5D-5L-questionnaire.

EQ-5D-5L DIMENSION		Month 0				Month 6			
		TD Group n (%)	F-F/D Group n (%)	Total n (%)	*p*-Value	TD Group n (%)	F-F/D Group n (%)	Total n (%)	*p*-Value
Mobility	No problems	215 (95.60)	150 (66.70)	365 (81.10)	0.00	216 (96.00)	181 (80.40)	397 (88.20)	0.00
	Slight problems	7 (3.10)	40 (17.80)	47 (10.40)		8 (3.60)	24 (10.70)	32 (7.10)	
	Moderate problems	2 (0.90)	23 (10.20)	25 (5.60)		1 (0.40)	16 (7.10)	17 (3.80)	
	Severe problems	1 (0.40)	10 (4.40)	11 (2.40)		0	4 (1.80)	4 (0.90)	
	Unable to	0	2 (0.90)	2 (0.40)		0	0	0	
Self-care	No problems	207 (92.00)	162 (72.00)	369 (82.00)	0.00	215 (95.60)	182 (80.90)	397 (88.20)	0.00
	Slight problems	13 (5.80)	33 (14.70)	46 (10.20)		10 (4.40)	26 (11.60)	36 (8.00)	
	Moderate problems	3 (1.30)	19 (8.40)	22 (4.90)		0	13 (5.80)	13 (2.90)	
	Severe problems	1 (0.40)	7 (3.10)	8 (1.80)		0	3 (1.30)	3 (0.70)	
	Unable to	1 (0.40)	4 (1.80)	5 (1.10)		0	1 (0.40)	1 (0.20)	
Usual activities	No problems	209 (92.90)	144 (64.00)	353 (78.40)	0.00	209 (92.90)	177 (78.70)	386 (85.80)	0.00
	Slight problems	11 (4.90)	41 (18.20)	52 (11.60)		14 (6.20)	31 (13.80)	45 (10.00)	
	Moderate problems	3 (1.30)	29 (12.90)	32 (7.10)		2 (0.90)	13 (5.80)	15 (3.30)	
	Severe problems	1 (0.40)	7 (3.10)	8 (1.80)		0	4 (1.80)	4 (0.90)	
	Unable to	1 (0.40)	4 (1.80)	5 (1.10)		0	0	0	
Pain/discomfort	No problems	124 (55.10)	95 (42.20)	219 (48.70)	0.02	139 (61.80)	148 (65.80)	287 (63.80)	0.32
	Slight problems	49 (21.80)	68 (30.20)	117 (26.00)		72 (32.00)	68 (30.20)	140 (31.10)	
	Moderate problems	34 (15.10)	44 (19.60)	78 (17.30)		10 (4.40)	5 (2.20)	15 (3.30)	
	Severe problems	16 (7.10)	13 (5.80)	29 (6.40)		4 (1.80)	4 (1.80)	8 (1.80)	
	Unable to	2 (0.90)	5 (2.20)	7 (1.60)		0	0	0	
Anxiety/depression	No problems	176 (78.20)	113 (50.00)	289 (64.20)	0.00	170 (75.60)	122 (54.20)	292 (64.90)	0.00
	Slight problems	29 (12.90)	71 (31.60)	100 (22.20)		43 (19.10)	88 (39.10)	131 (29.10)	
	Moderate problems	7 (3.10)	26 (11.60)	33 (7.30)		9 (4.00)	12 (5.30)	21 (4.70)	
	Severe problems	11 (4.90)	9 (4.00)	20 (4.40)		3 (1.30)	3 (1.30)	6 (1.30)	
	Unable to	2 (0.90)	6 (2.70)	8 (1.80)		0	0	0	
EQ-5D-5L-Utilities	Total (95CI)	0.89 [0.87; 0.91]	0.77 [0.74; 0.81]	0.83 [0.81; 0.85]	0.00	0.93 [0.92; 0.94]	0.87 [0.85; 0.90]	0.90 [0.89; 0.92]	0.00
EQ5D-VAS	Total (95CI)	76.22 [73.29; 79.15]	68.24 [65.10; 71.38]	72.23 [70.06; 74.40]	0.00	78.52 [75.68; 81.36]	73.32 [70.18; 76.46]	75.92 [73.80; 78.04]	0.03

F-F/D group: face-to-face dermatology; TD group: teledermatology.

**Table 3:** Health-related quality of life at 6 months.

Questionnaires	All (n = 450)				Teledermatology (n = 225)				Face-to-Face Dermatology (n = 225)			
Health-related Quality of Life-Specific												
	Baseline	Month 6	Differences	*p*-value	Baseline	Month 6	Differences	*p*-value	Baseline	Month 6	Differences	*p*-value
Skindex-29 Functionality	1.39 [1.17; 1.62]	0.27 [0.20; 0.34]	1.12 [0.92; 1.33]	0.00	0.85 [0.61; 1.09]	0.32 [0.2; 0.43]	0.54 [0.34; 0.74]	0.00	1.93 [1.57; 2.30]	0.23 [0.14; 0.31]	1.71 [1.36; 2.05]	0.00
Skindex-29 Symptoms	2.84 [2.63; 3.04]	1.30 [1.18; 1.43]	1.53 [1.36; 1.70]	0.00	2.21 [1.96; 2.47]	1.16 [0.98; 1.33]	1.05 [0.85; 1.25]	0.00	3.46 [3.16; 3.76]	1.45 [1.27; 1.63]	2 [1.76; 2.25]	0.00
Skindex-29 Emotions	3.40 [3.16; 3.65]	1.47 [1.34; 1.59]	1.94 [1.73; 2.15]	0.00	2.64 [2.33; 2.96]	1.34 [1.15; 1.52]	1.31 [1.06; 1.56]	0.00	4.16 [3.82; 4.51]	1.60 [1.43; 1.76]	2.57 [2.25; 2.88]	0.00
Total	7.64 [7.09; 8.20]	3.04 [2.79; 3.29]	4.59 [4.16; 5.02]	0.00	5.72 [5.07; 6.36]	1.34 [1.15; 1.52]	2.90 [2.46; 3.33]	0.00	9.56 [8.72; 10.39]	1.60 [1.43; 1.76]	6.28 [4.59; 5.60]	0.00
Health-related Quality of Life-General												
EQ-5D-5L VAS [95CI]	72.23 [70.06; 74.4]	75.92 [73.80; 78.04]	3.69 [2.90; 4.48	0.00	76.22 [73.29; 79.15]	78.52 [75.68; 81.36]	2.30 [1.43; 3.18]	0.00	68.24 [65.10; 71.38]	73.32 [70.18; 76.46]	5.08 [3.78; 6.38]	0.00
EQ-5D-5L utilities [95CI]	0.83 [0.81; 0.85]	0.90 [0.89; 0.92]	0.07 [0.06; 0.08]	0.00	0.89 [0.87; 0.91]	0.93 [0.92; 0.94]	0.04 [0.03; 0.05]	0.00	0.77 [0.74; 0.81]	0.87 [0.85; 0.90]	0.1 [0.08; 0.12]	0.00

**Table 4:** Changes in HRQoL between baseline and 6 months after the first visit, across Skindex-29 questionnaire.

Skindex-29	Month 0				Month 6			
	TD Group YES n (%)	F-F/D Group YES n (%)	Total YES n (%)	*p*-Value	TD Group YES n (%)	F-F/D Group YES n (%)	Total YES n (%)	*p*-Value
1. My skin hurts	45 (20.00)	108 (48.00)	153 (34.00)	0.00	10 (4.40)	17 (7.60)	27 (6.00)	0.233
2. My skin disease affects my sleep	23 (10.20)	39 (17.30)	62 (13.80)	0.04	1 (0.40)	4 (1.80)	5 (1.10)	0.372
3. I am worried that my skin disease might be something serious	142 (63.10)	151 (67.10)	293 (65.10)	0.429	20 (8.90)	38 (16.90)	58 (12.90)	0.016
4. My skin disease makes it difficult for me to do my work or hobbies	24 (10.70)	61 (27.10)	85 (18.90)	0.00	6 (2.70)	2 (0.90)	8 (1.80)	0.285
5. My skin disease affects my social life	33 (14.70)	60 (26.70)	93 (20.70)	0.002	8 (3.60)	6 (2.70)	14 (3.10)	0.787
6. My skin disease makes me depressed	37 (16.40)	75 (33.30)	112 (24.90)	0.00	24 (10.70)	82 (36.40)	106 (23.60)	0.00
7. My skin disease burns or stings	79 (35.10)	117 (52.00)	196 (43.60)	0.00	44 (19.60)	50 (22.20)	94 (20.90)	0.562
8. I tend to stay at home because of my skin disease	13 (5.80)	27 (12.00)	40 (8.90)	0.03	5 (2.20)	3 (1.30)	8 (1.80)	0.724
9. I worry that I will get scars from my skin disease	91 (40.40)	116 (51.60)	207 (46.00)	0.023	41 (18.20)	27 (12.00)	68 (15.10)	0.087
10. My skin itches	144 (64.00)	148 (65.80)	292 (64.90)	0.573	68 (30.20)	62 (27.60)	130 (28.90)	0.603
11. My skin disease affects my relationship with people I love	13 (5.80)	36 (16.00)	49 (10.90)	0.001	4 (1.80)	5 (2.20)	9 (2.00)	1
12. I am ashamed of my skin disease	44 (19.60)	37 (16.40)	81 (18.00)	0.462	4 (1.80)	4 (1.80)	8 (1.80)	1
13. I worry that my skin disease will get worse	149 (66.20)	180 (80.00)	329 (73.10)	0.001	150 (66.70)	120 (53.30)	270 (60.00)	0.005
14. I tend to do things alone because of my skin disease	19 (8.40)	24 (10.70)	43 (9.60)	0.522	10 (4.40)	2 (0.90)	12 (2.70)	0.036
15. I am angry about my skin disease	33 (14.70)	113 (50.20)	146 (32.40)	0.00	8 (3.60)	13 (5.80)	21 (4.70)	0.372
16. Water makes my skin disease worse (bathing, hand washing).	29 (12.90)	63(28.00)	92 (20.40)	0.00	6 (2.70)	2 (0.90)	8 (1.80)	0.285
17. My skin disease makes it hard for me to show my affection	9 (4.00)	42 (18.70)	51 (11.30)	0.00	7 (3.10)	9 (4.00)	16 (3.60)	0.8
18. My skin is irritated	81 (36.00)	146 (64.90)	227 (50.40)	0.00	56 (24.90)	94 (41.80)	150 (33.30)	0.00
19. My skin disease affects my relationship with others	17 (7.60)	38 (16.90)	55 (12.20)	0.004	11 (4.90)	5 (2.20)	16 (3.60)	0.202
20. My skin disease causes me to be embarrassed	16 (7.10)	29 (12.90)	45 (10.00)	0.058	6 (2.70)	1 (0.40)	7 (1.60)	0.122
21. My skin disease is a problem for the people I love	6 (2.70)	44 (19.60)	50 (11.10)	0.00	2 (0.90)	6 (2.70)	8 (1.80)	0.285
22. I am frustrated by my skin disease.	38 (16.90)	83 (36.90)	121 (26.90)	0.00	12 (5.30)	14 (6.20)	26 (5.80)	0.840
23. My skin is sensitive	99 (44.00)	152 (67.60)	251 (55.80)	0.00	71 (31.60)	100 (44.40)	171 (38.00)	0.006
24. My skin disease affects my desire to be with people	20 (8.90)	36 (16.00)	56 (12.40)	0.031	11 (4.90)	7 (3.10)	18 (4.00)	0.472
25. I find my skin disease humiliating	19 (8.40)	25 (11.10)	44 (9.80)	0.428	5 (2.20)	5 (2.20)	10 (2.20)	1
26. My skin disease bleeds	19 (8.40)	44 (19.60)	63 (14.00)	0.001	5 (2.20)	2 (0.90)	7 (1.60)	0.449
27. My skin disease makes me angry	26 (11.60)	128 (56.90)	154 (34.20)	0.00	31 (13.80)	55 (24.40)	86 (19.10)	0.006
28. My skin disease interferes with my sex life	9 (4.00)	15 (6.70)	24 (5.30)	0.294	5 (2.20)	1 (0.40)	6 (1.30)	0.216
29. My skin disease makes me tired	6 (2.70)	13 (5.80)	19 (4.20)	0.158	1 (0.40)	1 (0.40)	2 (0.40)	1

**Table 5:** Repeated measures design with group and time as fixed effects across EQ5D.

		F-F/D (n = 225)	Teledermatology (n = 225)	Group *p*-Value	Time *p*-Value	Group byTime *p*-Value
Mobility	Baseline	1.55 [1.43;1.67]	1;06 [1.02;1.10]	0.00	0.00	0.004
	Month 6	1.30 [1.21;1.39]	1.04 [1.01;1.07]			
UsualActivities	Baseline	1.60 [1.48;1.73]	1.11 [1.05;1.17]	0.00	0.00	0.002
	Month 6	1.31 [1.22;1.39]	1.08 [1.04;1.12]			
Self-care	Baseline	1.48 [1.36;1.60]	1.12 [1.06;1.18]	0.00	0.002	0.146
	Month 6	1.29 [1.20;1.38]	1.04 [1.02;1.07]			
Pain/ Discomfort	Baseline	1.96 [1.82;2.09]	1.77 [1.64;1.90]	0.274	0.00	0.028
	Month 6	1.40 [1.32;1.48]	1.46 [1.37;1.55]			
Anxiety/Depression	Baseline	1.77 [1.64;1.90]	1.37 [1.26;1.48]	0.00	0.005	0.099
	Month 6	1.54 [1.45;1.62]	1.31 [1.23;1.39]			
EQ5D-VAS	Baseline	68.24 [65.10; 71.38]	76. 22 [73.29; 79.15]	0.00	0.016	0.364
	Month 6	73.32 [70.18; 76.46]	78. 52 [75.68; 81.36]			
EQ-5D-5L-Utilities	Baseline	0.77 [0.74; 0.81]	0.89 [0.87; 0.91]	0.00	0.00	0.014
	Month 6	0.87 [0.85; 0.90]	0.93 [0.92; 0.94]			

F-F/D group: face-to-face dermatology.

**Table 6:** Repeated measures design with group and time as fixed effects across Skindex-29.

		F-F/D (n = 225)	Teledermatology (n = 225)	Group *p*-Value	Time *p*-Value	Group byTime *p*-Value
Skindex-29 Symptoms	Baseline	3.46 [3.16; 3.76]	2.21 [1.96; 2.47]	0.00	0.00	0.00
	Month 6	1.45 [1.27; 1.63]	1.16 [0.98; 1.33			
Skindex-29 Emotions	Baseline	4.16 [3.82; 4.51]	2.64 [2.33; 2.96]	0.00	0.00	0.00
	Month 6	1.60 [1.43; 1.76]	1.34 [1.15; 1.52]			
Skindex-29 Functionality	Baseline	1.93 [1.57; 2.30]	0.85 [0.61; 1.09]	0.00	0.00	0.00
	Month 6	0.23 [0.14; 0.31]	0.32 [0.2; 0.43]			
Total Skindex	Baseline	9.56 [8.72; 10.39]	5.72 [5.07; 6.36]	0.00	0.00	0.00
	Month 6	1.60 [1.43; 1.76]	1.34 [1.15; 1.52]			

F-F/D group: face-to-face dermatology.

## Data Availability

The datasets used and/or analyzed during the current study are available from the corresponding author on reasonable request.
